# Supplementary material for: Identification of driver genes and key pathways of non-functional pituitary adenomas predicts the therapeutic effect of STO-609
Source: PLoS One. 2020 Oct 29;15(10):e0240230. doi: 10.1371/journal.pone.0240230 (PMC7595405; doi:10.1371/journal.pone.0240230)
Supplement: Attachment — Submitted filename: Response to reviewers.docx [file pone.0240230.s002.docx]

Dear Editors and Reviewers:

Thank you for your letter and for the reviewers’ comments concerning our manuscript entitled “Identification of Driver Genes and Key Pathways of Non-functional Pituitary Adenomas Predicts the Therapeutic Effect of STO-609”. Those comments are all valuable and very helpful for revising and improving our paper, as well as the important guiding significance to our researches. We have studied comments carefully and have made corrections which we hope meet with approval. The main corrections in the paper and the responds to the reviewer’s comments are as following.

Reviewers' comments:

Reviewer 1:

An interesting paper, I think. To discover novel molecular targets for diagnosis and treatment for NFPA was the aim. Parameters which were investigated were DEGs (Differentially Expressed Genes), hub genes expression, enrichment functions and key pathways. The gene expression profiles of three datasets of were analyzed to identify the DEGs between NFPA tissue and normal tissue. Finally, the therapeutic effect of STO-609 was studied. It was shown that the enriched function and pathways were mainly related to Ca2+/CaM signaling pathway and parkinson’s disease. Therefore STO-609 was tested being a potent inhibitor regarding of the Ca2+/CaM signaling pathway.
A lot of characteristics of NFPA were determined. Then it is a little disappointing that the authors came up with only one targeting agent, the CaM kinase inhibitor, STO-609. On the other hand it seems that STO-609 has little toxicity (York et al Scientific reports 2017) [a].
Why was the non functional Pituitary adenomas (NFPA) aim of the study? The functionally active secrete pituitary hormones and cause clinical syndromes [b].
The NFPA are only detected when they are big. When would be the time to start diagnosis with the parameters investigated proposed? [c]
It was shown that 351 DEGs showed altered expression (compared to normal cells): 169 were up regulated and 182 were down regulated. Four hub genes were altered in expression and from this only STO-609 was tested. What other possibilities are possible to inhibit growth of NFPA? Can STO-609 be used for treatment even when NFPA are discovered late when they are big? [d]
The scratch assay is I think not very convincing: It is stated that “In Figure 4C, F, the width of scratched was significantly smaller after 24h in control group”. To me it seems that at the 24h time point the area in the control I wider than in the ST-609 treated plate, is not it? [e]

Response: Thanks for your comments. we are deeply impressed with your encyclopedic scholarship and conscientious attitude, and we are honored to get your comments.

a, Thanks for your comment. We have studied the report you mentioned carefully. The object of this study is providing potential biomarkers and drug targets for diagnosis and treatment of NFPAs, and verifying the anti-NFPAs effects of STO-609 preliminary. We proposed the possibility that STO-609 may be used to treat NFPAs. When come to the little toxicity of STO-609, we hold the view that we can’t give up eating for fear of chocking. Despite STO-609 has little toxicity, it still is one potential drug for NFPAs treatment. In addition, as inhibitor of CaMKK2, STO-609 had been proved playing roles in several diseases recently such as prostate cancer, gastric carcinoma, fracture, et al. Thus, STO-609 is still a potential drug.

b, NFPAs comprise approximately 30% of PA, and treatments for NFPAs are not satisfactory for both patients and doctors. The main methods to treat NFPAs were surgery and radiotherapy. However, tumors are difficult to be resected completely when invade the surrounding tissue. The recurrence rate after operation is up to 30%. In addition, the effective drug is short for NFPAs treatment so far. So we can tell novel therapeutic methods and medicine are needed for NFPAs. In this study, we not only verified the therapeutic effect of STO-609 in Non-functional Pituitary Adenomas (NFPAs), but also provide DEGs and enrichment pathways which could also be applied for inhibit growth of NFPA as drug targets.
c, Thanks for your comments. It is a professional and wise question. In our study, all the DEGs we provided were potential biomarkers for diagnosis and prognosis, as well as targets for therapy. When come to diagnosis, those DEGs may be applied as tumor markers for people who had a high risk when they take periodic physical examination, and applied to monitor recurrence of NFPA after surgery. So, it is not our focus about when to start diagnosis.

d, Thank you for professional and rigorous comments. In this study, we not only verified the therapeutic effect of STO-609 in Non-functional Pituitary Adenomas (NFPAs), but also provide DEGs and enrichment pathways which could also be applied for inhibit growth of NFPA as drug targets. As you mentioned, the effect of STO-609 on big NFPA is not clear now, and we will conduct animal experiments for further study in future.

e, Thank you for reminding this omission, the Figure had been corrected.

Reviewer 2:

The authors presented bioinformatics and experimental work on Non-functional Pituitary Adenomas.
I would advise the authors to imrpove the english in the manuscript. Idealy to send it to native speaking enlish lector.
I Will point out some sentences that sounded strange to me:
- line 123: For three samples or for three datasets?
- line 199: ...were screened out... Please change the sentence to make it more understandable
- line 127: The KEGG analysis showed the most significantly enriched pathways. The sentence has to include the pathways.
- line 224: The hub nodes with degrees more than 12 were screened using the STRING. Maybe with degrees you mean interactions? And again there is screened. Plase revise the sentence. [a]

Please revise the manuscript were there are similar sentences than described above.
Also I would like the authors to revise: when writing about hub genes. The hubs in STRING are proteins not genes, because this is PPI netowork - protein pprotein interaction network. The authors are mixing the terminology and abbreviations. When talking about proteins the abbreviations are normal, when talking about genes, the abbreviations are in italic. [b]
- line 293/294 should be: Four hub proteins encoded by genes (genes in italic - CALM1, PRDM10, RIPK4 and MAD2L1) had never been reported related to NFPA.
Please revise all similar sentences. [c]

Response: Thank you for your excellent comments, they benefit us a lot, we are honored to get your comments. This manuscript had been polished by a native English speaker.

a, Thanks for your comments. All the sentence you put out had been polished.

b, Thanks for your professional comments. All the abbreviations in this manuscript had been corrected, and now they are in italic.

c, All similar sentences had been revised in this manuscript.

Dear Editors,

Thank you for your work dealing with my manuscript, thank you very much. If there is any problem, please don’t hesitate to contact us. I will reply you as soon as possible. Best wishes to you. May its blessings lead into a wonderful year for you and all whom you love.
