# Supplementary material for: Identification of driver genes and key pathways of non-functional pituitary adenomas predicts the therapeutic effect of STO-609
Source: PLoS One. 2020 Oct 29;15(10):e0240230. doi: 10.1371/journal.pone.0240230 (PMC7595405; doi:10.1371/journal.pone.0240230)
Supplement: Attachment — Submitted filename: Response to Reviewers .docx [file pone.0240230.s003.docx]

Dear Editors and Reviewers:

Thank you for the letter and the reviewers’ comments concerning our manuscript entitled “Identification of Driver Genes and Key Pathways of Non-functional Pituitary Adenomas Predicts the Therapeutic Effect of STO-609”. Those comments are all valuable and helpful for revising and improving our paper, as well as the important guiding significance to our researches. We have studied these comments carefully and have made corrections which we hope meet with approval. The main corrections in the paper and the responds to the reviewer’s comments are as following.

Reviewers' comments:

Reviewer 1:

In this manuscript, the authors aim to identify novel molecular pathways and genes regulating non-functional pituitary adenomas (NFPA) which are usually benign tumors, to identify therapeutic targets. They utilize three GEO data sets from gene expression profiling of non-invasive and invasive NFPAs to perform data mining analyses to identify differentially expressed genes, hierarchical clustering, gene ontology, hub gene expression, biological function and pathway analyses. From these analyses, they identify CALM1, PRDM10, and MAD2L1 were recognized as hub genes upregulated in invasive NFPAs while RIPK4 was identified as a downregulated hub gene. Since CALM1, the gene encoding for the intracellular Ca2+ receptor calmodulin (CaM), is one of the upregulated hub genes, the authors utilize STO-609, a selective inhibitor of CaMKKs, the upstream regulator of the CaMK signaling cascade that is regulated by Ca2+/CaM binding, to modulate CaM function in one human NFPA cell line and one mouse pituitary adenoma cell line. They report that treatment of these cells with STO-609 results in a loss of cell viability and migration potential. This is an interesting story, where the data mining and the identification of the four hub genes and a potential similarity to Parkinson disease are interesting. However the manuscript suffers from some very serious flaws:

The whole paper is poorly written with bad sentence construction, grammatical and spelling mistakes, wrong word usage and use of colloquial phrases such as “It’s”, “what’s more”, etc. The impact of the potentially exciting discovery of the data mining results they describe for figures 1-3 is lost due to the poor English writing. The authors should extensively edit the paper for English language [a].

There are many abbreviations in this paper, which makes it hard to follow the content at times. Authors should include a table of all the abbreviations used in the paper [b].

The rationale for the STO-609 studies is not clear at all. This should be made in the last paragraph of the introduction as well as in the results section which describes these studies. Moreover, STO-609 does not affect the function of Calmodulin. A statement that STO-609 blocks CALM1 function appears repeatedly in the text in the introduction and discussion without any references to back this claim. This reviewer is not aware of any studies showing this in the literature. If the authors have evidence, they should cite the appropriate papers to back up this claim [c].

Nevertheless, the authors are justified in testing STO-609 against NFPAs. However, this needs to be well-justified [d].

None of the figures are legible. All figures appear blurry, pixelated, and I could not understand what any of them show. Given this, it was hard to judge this paper [e].

Specifically, in figure 4A and B, none of the X-axis values which indicate STO-609 concentrations used for the dose-response curve on cell viability, are legible. So I am not able to judge if the treatment worked or not [f].

The authors do not indicate how STO-609 was solubilized, and prepared for the in vitro assays. They talk about a “solvent DMSO control” in the Materials and Methods section but never show this in Figure 4. Thus, it is hard to understand whether the effects they see of STO-609 on these cells is due to solvent toxicity or STO-609 [g].

When performing experiments to understand the effects of a drug on cancer cells, it is important to test if that drug does the same to normal cells. The authors should test STO-609 on normal pituitary cells. There is such a cell line available from the ATCC – AtT-20 mouse normal pituitary cells [h].

Discussion makes several statements and claims without any references. Also, the potential link between Parkinson’s disease and NFPA would be interesting. But since none of the figures are legible, it is not possible to reach this conclusion from the data provided. Moreover, the discussion of this connection should be written in a more focused manner [i].

Table 2: What do the authors mean by the column titled “Betweenness”? This is an incorrect term anyways [j].

Response: Thanks for your comments. We are deeply impressed with your encyclopedic scholarship and conscientious attitude, and we are honored to get your comments.

a, Thanks for your professional comments. This manuscript was polished by a native English speaker, and inopportune expressions was corrected.

b, We had provided the Abbreviations document. Thanks for your comments.

c, Thanks for your professional comments. As we described in this manuscript, STO-609 was an inhibitor of Ca2+/calmodulin-dependent protein kinase kinase (CaM-KK2), which is an important downstream role of Calmodulin. So, STO-609 affect the function of Calmodulin indirectly. The statement that STO-609 blocks CALM1 function in this manuscript was expressed more accurate, and the rationale for the STO-609 studies also was made more clear in the last paragraph of the introduction as well as in the results section which describes these studies. Thanks for your comments again.

d, In order to verify the anti-NFPAs effects of STO-609, we performed a series of assays such as CCK-8 assay, Colony-forming assay, in vitro scratch assay and apoptosis assays. CCK-8 and colony-forming assay were conducted to estimate whether STO-609 inhibited the proliferation of NFPA cells, while in vitro scratch assay could demonstrate whether STO-609 reduced the migration of NFPA cells. In addition, apoptosis assays revealed how STO-609 induced the apoptosis of NFPA cells. These experimental methods were reliable and widely used in studying the effects of drugs on cells. For example, Zhang W, Lei Z etc performed CCK-8, colony-forming assay and cell scratch assay to research the effect of water extract of sporoderm-broken spores of ganoderma lucidum on osteosarcoma cells (Zhang W, Lei Z, Meng J, Li G, Zhang Y, He J, Yan W. Water Extract of Sporoderm-Broken Spores of Ganoderma lucidum Induces Osteosarcoma Apoptosis and Restricts Autophagic Flux. OncoTargets and Therapy 2019;12:11651-11665. doi: 10.2147/OTT.S226850.). The details of assays in vitro were provided and Figures was polished according to your following comments. Thanks for your professional comments.

e, Sorry for blurry figures. All the figures was replaced with clear PDF versions.

f, Sorry for that. We optimized the format of Figure 4, and made it easier to read. Thanks for your comments.

g, Thanks for your comments. Firstly, we dissolved STO-609 in pure DMSO to obtain the mother liquor, then we respectively added appropriate culture medium to the mother liquor to configure cell culture medium with STO-609 concentration of 0.25μmol/L and 1μmol/L. The concentration of DMSO was less than 0.5%. In this concentration, the influence of DMSO on cells was negligible, therefore we decided not to set up the DMSO control group. This statement was added to the manuscript.
h, Thanks for your comments. The ATCC – AtT-20 mouse normal pituitary cells were tested in this manuscript, and the results were added in this manuscript.

i, Thanks for your professional comments. The reference you mentioned was added in discussion. In this study, GSEA analysis was performed for further insight of DEGs, and the results showed that the pathway of Parkinson’s disease (PD) was significantly altered. That implicit the potential link between PD and NFPA. Thus, we hypothesized that NFPA and PD might be homologous and NFPA patients may take a rather higher risk suffering PD. This hypothesis needed further studies to elucidated in future.

j, Thank you for pointing out our mistake. We changed “Betweenness” to “Betweenness Centrality” in **Table 2***.*

Reviewer 2:

Despite that, the subject is very interesting but the presented studies are weakly performed and described. Therefore it is difficult to agree with the authors' conclusions in the present form of the manuscript.

Why authors choose only one Ca2+/CaM kinase inhibitor? Its cause that results are very unbelievable [a].

The authors omitted a description of cell treatment, probably, therefore, the concentrations of agent added to the cell culture is not described in the materials and methods [b].

The authors did not describe the surface of the dishes used in the colony-forming assay. [c] Additionally, the results of that experiment are presented unreadable. Therefore it is impossible to assess how many colonies grown up? The authors should present the absolute colony numbers per plate (with plate surface) or per cm2. Did the authors check the percentage of grown colonies in control? If it is less than 50% then the cells are not suitable for this assay. [d] Additionally, the figures (4B) presented cell treated with 0.25 mikrom/L looks similar to 1 mikrom/L. Where did the authors saw the differences presented in the graphs? [e] Similar 4C - the pictures do not presented the results showed in the graphs [f].

In those reasons demonstrated studies are not convincing and required future analysis that could confirm the authors' supposition.

Response: It’s our honor to obtain your comments, and thank you for your excellent comments that benefit us a lot.

a, Thanks for your comments. This study aims to provide potential biomarkers and drug targets for diagnosis and treatment of NFPAs and verify the anti-NFPAs effects of STO-609 preliminary. Actually, we compared all Ca2+/CaM kinase inhibitors, but chose STO-609 finally because STO-609 had been proved playing roles in several diseases recently such as prostate cancer, gastric carcinoma, et al. Thanks for your comments again.

b, Thanks for your comments. The description of STO-609 concentrations was added in this manuscript.

c, Thanks for your professional comments. In colony-forming assay, we used a six-well cell culture plate rather than petri dishes, and the surface area of each well of the culture plate was 9.6 cm^2^. This description was added in this manuscript.

d, Thanks for your professional comments. We reanalyzed the results of colony-forming assay, and added the absolute colony numbers (with plate surface) to the manuscript and Figure 4. In addition, the percentage of grown colonies in control group was 70%.

e, The figure and graphs were replaced with clear PDF versions. Thanks for your comments which benefit us a lot.

f, Thanks for your comments. The figure was polished.

Dear Editors,

Thank you for your work dealing with my manuscript, thank you very much. If there is any problem, please don’t hesitate to contact us. I will reply you as soon as possible. Best wishes to you. May its blessings lead into a wonderful year for you and all whom you love.
