# Supplementary material for: Identification of driver genes and key pathways of non-functional pituitary adenomas predicts the therapeutic effect of STO-609
Source: PLoS One. 2020 Oct 29;15(10):e0240230. doi: 10.1371/journal.pone.0240230 (PMC7595405; doi:10.1371/journal.pone.0240230)
Supplement: Attachment — Submitted filename: Response to reviewers.docx [file pone.0240230.s004.docx]

1. If the authors have adequately addressed your comments raised in a previous round of review and you feel that this manuscript is now acceptable for publication, you may indicate that here to bypass the “Comments to the Author” section, enter your conflict of interest statement in the “Confidential to Editor” section, and submit your "Accept" recommendation.

Reviewer #1: (No Response)

Reviewer #2: (No Response)

2. Is the manuscript technically sound, and do the data support the conclusions?

The manuscript must describe a technically sound piece of scientific research with data that supports the conclusions. Experiments must have been conducted rigorously, with appropriate controls, replication, and sample sizes. The conclusions must be drawn appropriately based on the data presented.

Reviewer #1: Yes

Reviewer #2: (No Response)

3. Has the statistical analysis been performed appropriately and rigorously?

Reviewer #1: (No Response)

Reviewer #2: No

Response: The statistical analysis was updated. Independent-samples t test was conducted to analyze quantitative data of bioinformation analysis, while Analysis of Variance (ANOVA) was performed to analyse multiple comparison data of cell experiments. Besides, we conducted Dunnett-t test as post hoc test after ANOVA. We confirmed that the level of significance was P < 0.05. Thanks a lot for the valuable comments.

4. Have the authors made all data underlying the findings in their manuscript fully available?

The PLOS Data policy requires authors to make all data underlying the findings described in their manuscript fully available without restriction, with rare exception (please refer to the Data Availability Statement in the manuscript PDF file). The data should be provided as part of the manuscript or its supporting information, or deposited to a public repository. For example, in addition to summary statistics, the data points behind means, medians and variance measures should be available. If there are restrictions on publicly sharing data—e.g. participant privacy or use of data from a third party—those must be specified.

Reviewer #1: Yes

Reviewer #2: Yes

5. Is the manuscript presented in an intelligible fashion and written in standard English?

PLOS ONE does not copy edit accepted manuscripts, so the language in submitted articles must be clear, correct, and unambiguous. Any typographical or grammatical errors should be corrected at revision, so please note any specific errors here.

Reviewer #1: Yes

Reviewer #2: No

Response: Thanks for the professional comments. We had already re-polished the language in this manuscript to meet the requirements of PLOS ONE.

6. Review Comments to the Author

Please use the space provided to explain your answers to the questions above. You may also include additional comments for the author, including concerns about dual publication, research ethics, or publication ethics. (Please upload your review as an attachment if it exceeds 20,000 characters)

Reviewer #1: The manuscript has improved a lot with the revision. A few minor concerns remain which need to be addressed.

1. Pages 5-6, lines 128-129: - This sentence needs to be revised. The reference # 49 does not show that STO-609 blocks CALM1 function. You could say, for instance, that you wanted to use STO-609 to block CaMKK signaling, as it operates downstream of CALMA. Please understand that calmodulin activates many proteins, and CaMKK is only one of them.

Response: Thanks a lot for your detailed comments. This sentence was revised according to this suggestion.

2. Page 7 line 177 - Delete the part of the sentence that says STO-609 blocks CALM1 gene function. It does not. Also you need to say here how you made the STO-609 solution.

Response: This part was revised according to your comments. Thank you for the valuable suggestions.

3. Page 8, line 197-198: - Change "mother liquor" to stock solution. The description of STO-609 stock preparation should be moved earlier to page 7 line 177 - first time you mention about using STO-609 on cells.

Page 8, lines 199-202: I disagree. Even 0.05% DMSO could have an effect on cells. This is an important control. Also, Remove the sentence: "different doses of STO-609 were used to treat those cells".

Page 11, lines 268 -276: It is still not clear why they chose to block CaMKK function using STO-609 while PRDM10 scored the highest.. Please make this clear. You do have a paragraph in the Discussion - page 14 lines 348-350 that provide a good rationale. This can be utilized in the Introduction and the Results sections to provide the rationale for blocking CaMKK.

Pages 11-12, lines 281-282: - Revise these sentences: Meanwhile, only tiny decline was observed in STO-609 group. What’s more, the wounds in the drug group were quite wider than in control group after 48h.

Response: These sections were all revised with your comments. Besides, the point of view that the influence of 0.1% DMSO on cells is negligible is supported by researches as following:

[1] Qi W, Ding D, Salvi R J. Cytotoxic effects of dimethyl sulphoxide (DMSO) on cochlear organotypic cultures[J]. Hearing Research, 2008, 236(1-2):52-60
[2] Nirogi R, Kandikere V, Bhyrapuneni G, et al. Effect of dimethyl sulfoxide on in vitro cytochrome P4501A2 mediated phenacetin O-deethylation in human liver microsomes[J]. Drug Metabolism & Disposition the Biological Fate of Chemicals, 2011, 39(11):2162

These suggestions benefited us a lot in improving this manuscript. It is our great honor to obtain your valuable and professional comments.

Reviewer #2: Unfortunately, despite the author's corrections, the manuscript still has low quality.[a] The authors' explanation about the reason for choosing the only one inhibitor in the present studies is very unclear. The conclusions from such limited analysis are dubious.[b] The quality of figures and language make reading the text difficult.[c] The result of wound healing is still inconsistent (graphs and images) - Figure 4.[d]

To sum up, I regret to inform you that I do not recommend the manuscript in the present form for publication in PLOS ONE.[a]

Response: a, In this study, bioinformatical methods were conducted to identify DEGs, comprehensively investigate hub genes, annotate enrichment functions and key pathways of NFPAs, and performed a series of cell assays to verify the therapeutic effect of STO-609 on NFPAs. The bioinformatical methods and cell assays in this study were scientific and reliable, which were widely used in researches as following:

[1] Zhong S, Wu B, Li J et al. T5224, RSPO2 and AZD5363 are novel drugs against functional pituitary adenoma [J]. Aging, 2019;11(20):9043-9059.
[2] Zhong S, Wu B, Han Y et al. Identification of Driver Genes and Key Pathways of Pediatric Brain Tumors and Comparison of Molecular Pathogenesis Based on Pathologic Types [J]. World Neurosurgery, 2017;107:990-1000.
[3] Shen S, Kong J, Qiu Y et al. Identification of core genes and outcomes in hepatocellular carcinoma by bioinformatics analysis [J]. Journal of Cellular Biochemistry, 2019;120(6):10069-10081.

b, Thanks for your comments. This study aims to provide potential biomarkers and drug targets for diagnosis and treatment of NFPAs and verify the anti-NFPAs effects of STO-609 preliminary. After comparing all Ca2+/CaM kinase inhibitors, we found STO-609 was a selective and cell-permeable inhibitor of the CaM-KK, and was proved playing roles in prostate cancer, gastric carcinoma, et al. We conjectured STO-609 was also a potential drug for NFPAs, therefore we regarded STO-609 as the major object in this research. Other Ca2+/CaM kinase inhibitors were outside the scope of this study. We believe other Ca2+/CaM kinase inhibitors could be interesting directions in further researches.

c, This language in this manuscript was polished to meet the requirements of PLOS ONE. Besides, we improved the resolution of figures to make them more clear for publication. Thanks for your comments.

d, We increased the images resolution of the microscopy images in Fig.4 and believed that this version was clear enough to compare the widths of scratch. Thank you for the useful suggestions.

Dear editors,

Thanks sincerely for your hard work dealing with this manuscript. These professional comments and suggestions really helped us a lot to improve the quality of this manuscript. We believe these opinions will benefit us on our researches in the future.

If there is any problem, please don’t hesitate to contact us. We will reply you as soon as possible. Best wishes to you and all whom you love.
